# Discovery of a Vertebral Skeletal Stem Cell Driving Spinal Metastases

**DOI:** 10.21203/rs.3.rs-2106142/v1

**Published:** 2023-01-25

**Authors:** Jun Sun, Lingling Hu, Seoyeon Bok, Alisha R Yallowitz, Michelle Cung, Jason McCormick, Ling J Zheng, Shawon Debnath, Yuzhe Niu, Adrian Y Tan, Sarfaraz Lalani, Kyle W Morse, Daniel Shinn, Anthony Pajak, Zan Li, Na Li, Ren Xu, Sravisht Iyer, Matthew B Greenblatt

**Affiliations:** 1 Department of Pathology and Laboratory Medicine, Weill Cornell Medicine, New York, NY, USA; 2 Flow Cytometry Core Facility, Weill Cornell Medicine, New York, NY, USA; 3 Genomics Resources Core Facility, Weill Cornell Medicine, New York, NY, USA; 4 Department of Spine Surgery, Hospital for Special Surgery, New York, NY, USA; 5 Weill Cornell Medical College, 1300 York Avenue, New York, NY 10065, USA; 6 State Key Laboratory of Cellular Stress Biology, School of Medicine, Xiamen University, Xiamen, Fujian, China.; 7 Research Division, Hospital for Special Surgery, New York, NY, USA

**Keywords:** Stem cell, Bone, Vertebrae, Metastasis, Osteoblast

## Abstract

Vertebral bone is subject to a distinct set of disease processes from those of long bones, notably including a much higher rate of solid tumor metastases that cannot be explained by passive blood flow distribution alone. The basis for this distinct biology of vertebral bone has remained elusive. Here we identify a vertebral skeletal stem cell (vSSC), co-expressing the transcription factors ZIC1 and PAX1 together with additional cell surface markers, whose expression profile and function are markedly distinct from those of long bone skeletal stem cells (lbSSCs). vSSCs display formal evidence of stemness, including self-renewal, label retention and sitting at the apex of their differentiation hierarchy. Lineage tracing of vSSCs confirms that they make a persistent contribution to multiple mature cell lineages in the native vertebrae. vSSCs are physiologic mediators of spine mineralization, as genetic blockade of the ability of vSSCs to generate osteoblasts results in defects in the vertebral neural arch and body. Human counterparts of vSSCs can be identified in vertebral endplate specimens and display a conserved differentiation hierarchy and stemness. Multiple lines of evidence indicate that vSSCs contribute to the high rates of vertebral metastatic tropism observed clinically in breast cancer. Specifically, when an organoid system is used to place both vSSCs and lbSSCs in an identical anatomic context, vSSC-lineage cells are more efficient than lbSSC-lineage cells at recruiting metastases, a phenotype that is due in part to increased secretion of the novel metastatic trophic factor MFGE8. Similarly, genetically targeting loss-of-function to the vSSC lineage results in reduced metastasis rates in the native vertebral environment. Taken together, vSSCs are distinct from other skeletal stem cells and mediate the unique physiology and pathology of vertebrae, including contributing to the high rate of metastatic seeding of the vertebrae.

We recently reported that periosteal and endosteal bone is formed by different stem cell populations, which, alongside the work of others, indicated that bone is not formed by a single stem cell, but rather by a diverse set of stem cells, each with signature anatomic locations and functions^1–3^. Finding that the distinct physiology of different regions within a single bone is due to the presence of distinct stem cells raised the further question of whether the differences in biology between different bones could also be due to as of yet undiscovered stem cells. Based on this, we hypothesized that the vertebrae were likely to be formed from a distinct skeletal stem cell given that both the evolutionary history and developmental ontology of the vertebrae are markedly different than those of the limb bones^4^. This is supported by the observation that lineage tracing using *Prrx1-cre* provides robust labeling of long bones but not vertebrae (Extended Data Fig. 1a). The axial neural crest, notochord and muscle stem cells also do not contribute to vertebral bone (Extended Data Fig. 1). To identify specific markers of vertebral stem cells (vSSC), we used the observation that skeletal stem cells (SSCs) at multiple anatomic sites, including calvarium, long bone periosteum and endosteum appear to share a core set of surface markers, all being Lineage-CD200+CD105-THY1-6C3- cells^1–3^. To identify candidate genes that would, in combination with this immunotype, uniquely mark candidate vSSCs, cells bearing this immunophenotype were sorted from vertebrae and long bones (long bone SSCs, lbSSCs) and subjected to transcriptional profiling. This transcriptional profiling revealed that candidate vSSCs and lbSSCs displayed broadly different transcriptional profiles, supporting that candidate vSSCs represent a distinct cell type (Fig. 1a, Extended Fig. 2a).

Before using this data to identify vSSC specific genes, this transcriptome analysis was also used to refine the SSC immunophenotypic definition, identifying EMBIGIN (EMB) as a marker of mature osteoblasts and maturing chondrocytes contaminating current SSC definitions (Fig. 1). Recent evidence indicates that a substantial number of cells identified as SSCs based on immunophenotype (Lin-CD200+CD105-THY1-6C3- cells^1–3^) express osteocalcin, a signature marker of mature osteoblasts^5^. Similarly, expression of CD200 has also been identified on mature osteoblasts^6^. Based on this data, we sought additional markers that could be used to identify and exclude these mature cells contaminating current SSC definitions. Cell surface markers expressed in candidate vSSCs and lbSSCs were screened, ultimately finding that EMB marked a distinct subset of both populations (Fig. 1b). EMB has been previously noted as a marker of skeletal populations in the marrow cavity, though the identity of EMB+ cells was unknown^7^. Transcriptome analysis shows the EMB+ fraction of SSCs expresses much higher levels of osteoblast markers including *Alpl*, *Spp1*, *Runx2*, and *Sp7*/*osterix*, and hypertrophic chondrocyte markers including *Col10a1* and *Ihh*, than the EMB− fraction (Fig. 1c, d). In line with this, immunostaining validated that both mature osteoblasts and hypertrophic chondrocytes express EMB (Fig. 1e, Extended Data Fig. 2b, c). Additionally, FACS of *Osteocalcin-cre* (*Ocn-cre*) *mTmG* mice identifies a very high degree (mGFP+EMB+/Total mGFP+>90%) of concordance between the EMB+ fraction of vSSCs and lbSSCs and the *Ocn-cre* mGFP+ fraction of cells (Fig. 1f). In transplantation studies, the EMB-fraction of candidate vSSCs was able to generate the EMB+ fraction of vSSCs, but the EMB+ fraction was not able to generate the EMB− fraction of vSSCs (Fig. 1g), demonstrating that the EMB+ fraction of SSCs does not sit at the apex of their differentiation hierarchy. Thus, EMB identifies mature cell types, including osteoblasts and hypertrophic chondrocytes, contaminating current stem cell immunophenotypic definitions and thereby refines the definition of SSCs. Hereafter candidate vSSCs will be defined as Lin-CD200+CD105-THY1-6C3-EMB- cells that are further fractionated based on genetic lineage reporters.

Transcriptional profiling identified a set of genes that showed selective or differential expression in postnatal candidate vSSCs but not lbSSCs. Notably, this included a number of genes reflecting the somitic origins of the vertebrae such as *Pax1, Zic1*, *Pax9* and *Prdm6*^8*–*10^, indicating that transcriptional signatures associated with the unique embryonic developmental origin of the vertebrae are carried forward postnatally (Extended Data Fig. 3a). Next, genes comprising a specific transcriptional “signature” for vSSCs that can be followed as a mark of vSSC identity were established, with *Dpt* and *Ramp2* showing robust differential expression in vSSCs versus lbSSCs (Extended Data Fig. 3b–d). Next, among the genes selectively expressed in vSSCs, we additionally sought genes that could be used to construct cre lines selectively targeting vSSCs, focusing on transcription factors (Fig. 2a, Extended Data Fig. 3d). These transcription factors were screened for their ability to drive expression of vSSC transcripts when their expression was enforced in lbSSCs (Extended Data Fig. 3d). Through this, we found that *Zic1* (Zinc finger in cerebellum 1), which is best known as a transcription factor regulating cerebellar development^11,12^, displayed highly specific expression in candidate vSSCs but not lbSSCs. *Zic1* was able to enforce expression of the vSSC-specific markers, including *Dpt* and *Ramp2* (Extended Data Fig. 3d). The transcription factor *Pax1* was also prioritized for further evaluation based on both displaying highly selective expression in vSSCs and not lbSSCs and longstanding observations that “undulated” mice with spontaneous mutations in *Pax1* display vertebral defects^13–15^.

Based on this, a *Zic1-cre* and a *Pax1-creER*^*T2*^ were constructed, finding that both provide selective labeling of vertebrae when bred to the *mTmG* reporter allele (Fig. 2, Extended Data Fig. 4, 5). Within the skeleton, *Zic1-cre* provided absolute specificity for vertebrae versus long bones, with no detectable long bone signal (Fig. 2b, c). Consistent with vSSCs being Zic1-positive, *Zic1-cre* labeled multiple skeletal lineages, including endplate cartilage, osteoblasts, marrow adipocytes and the annulus fibrosis surrounding the intervertebral disc (Fig. 2b, d, e). Similar labeling was observed throughout the spinal column, from cervical to sacral vertebrae (Fig. 2f). Flow cytometry showed a corresponding diversity in *Zic1*-lineage cells, with *Zic1-cre* labeling candidate vSSCs in addition to other populations similar to reported non-stem populations in long bones, such as CD105+ or THY1+ cells (Extended Data Fig. 5a).

For *Pax1*, a *creER*^*T2*^ strategy was selected to avoid any labeling observed being due to the known expression of *Pax1* in the developmental precursors of the vertebrae^16^. Pulse-chase labeling in *Pax1-creER*^*T2*^ mice demonstrated that initial labeling occurred in the vertebral endplate cartilage, with subsequent spread of the labeled clones through the proliferating cartilage columns over the subsequent month, ultimately leading to labeling of subchondral osteoblasts (Fig. 2g, Extended Data Fig. 5c). This pattern of labeling indicates that the most immature *Pax1*-lineage cells localize to the resting zone of the endplate cartilage and progress through proliferative and hypertrophic chondrocytes before generating osteoblasts, paralleling observations made regarding lbSSCs^2,17^. This physical progression of *Pax1-creER*^*T2*^ labeled cells from the resting zone of the endplate, through proliferating and hypertrophic chondrocytes to osteoblasts also paralleled a differentiation sequence observed by flow cytometry, with initial labeling of EMB− vSSC populations that over time progressed to labeling of EMB+CD200+ mature cells (Fig. 2h). Immunofluorescence staining of *Zic1-cre*-labeled populations for PAX1 demonstrated that the majority of endplate *Zic1*-lineage cells at the resting zone are also PAX1 positive (Fig. 2i). Similarly, FACS-isolated *Zic1*-lineage vSSCs, but not vertebral hematopoietic lineage cells nor lbSSCs, displayed robust co-expression of *Zic1* and *Pax1* (Extended Data Fig. 5e). Thus, multiple lines of evidence indicate that, with reference to immunophenotypic vSSCs, *Zic1* and *Pax1* label highly overlapping populations, therefore these two mouse strains offer complementary approaches to examine a *Pax1*+*Zic1*+ candidate vSSC.

Given this evidence, the stemness of these candidate vSSC was next interrogated, finding that serial transplantation, *in vivo* clonality assays and label retention studies all converge on vSSCs representing true stem cells. First, the clonality of the contribution of *Pax1*-lineage vSSCs to vertebral cellularity was confirmed using *Pax1-creER*^*T2*^; *Rosa26*^*confetti*^ mice, observing a clonal contribution similar to that reported for long bone SSCs using a similar system^2,3^, which was particularly evident in proliferating chondrocytes (Fig. 3a). Chromatin label retention due to the slow proliferative cycling of stem cells is often associated with stemness *in vivo*^18,19^. In line with this, label retention studies using both “tet-off” (*R26-M2rtTA; TetOP-H2B-GFP*) and “tet-on” (*R26-tTA; TetOP-H2B-GFP*) pulse chase systems revealed preferential label retention within the vertebrae after a 6-month chase period in a *Pax1*+ population within the cartilage endplate (Fig. 3b–d, Extended Data Fig. 6). Flow cytometry showed that these label retaining cells are vSSCs (Fig. 3c, Extended Data Fig. 6). This simultaneous flow cytometry and imaging analysis further supports the localization of vSSCs to the resting zone of the cartilage endplate. Due to the lower efficiency of labeling with the inducible *Pax1-creER*^*T2*^ system, *Zic1-cre* was used for experiments requiring isolation of vSSCs. First, FACS isolated *Zic1*-lineage vSSCs formed bone organoids after transplantation, a functional signature of SSCs ^1,3,20^ (Fig. 3e). Similar to lbSSCs, vSSC-derived organoids included bone matrix-forming osteoblasts, an internal marrow space including both host-derived hematopoietic cells and graft-derived marrow adipocytes, and cartilage, demonstrating the *in vivo* multipotency of *Zic1*-lineage vSSCs (Fig. 3f–i). Additionally, *Zic1*-lineage vSSCs, but not *Zic1*-lineage EMB+CD200+ cells were uniquely able to both self-renew and reconstitute their entire lineage after transplantation (Fig. 3j, k, Extended Data Fig. 7a). Moreover, *Zic1*-lineage vSSCs reisolated after the first round of transplantation were capable of again reconstituting the entire *Zic1*-lineage, demonstrating that the *Zic1*-lineage vSSCs present after the first round of transplantation do not merely retain their immunophenotype, but moreover retained their original differentiation potential (Fig. 3j, k, Extended Data Fig. 7a). Taken together, vSSCs display all the major stemness features demonstrated for lbSSCs^1–3^.

Ablating the contribution of *Zic1*-lineage vSSCs to the pool of bone forming osteoblasts demonstrates their physiologic importance for vertebral bone formation. Conditional deletion of *Osterix* (*Sp7*), a transcription factor absolutely required for osteoblast differentiation^21^, with *Zic1-cre* selectively blocks the contribution of *Zic1*-lineage vSSCs to bone formation. The resulting *Zic1-cre Osx*^*fl/fl*^ mice display severe vertebral defects, including paraplegia with 100% penetrance, starting from 2 weeks of age due to spine instability. This corresponded to severe kyphoscoliosis and an absence of the majority of the dorsal neural arch (Fig. 4a–c, Extended Data Fig. 8a–c). Additionally, the vertebral body was substantially impacted, with an approximately 50% reduction in bone mass within the vertebral body (Fig 4. d, e). As the paresis associated with the severe spinal instability phenotype of *Zic1-cre Osx*^*fl/fl*^ mice could potentially confound assessment of bone mass, we also sought to generate mice with a less severe attenuation of *Zic1*-lineage vSSC osteogenic capacity. STAT3 is a positive regulator of osteoblast differentiation ^22,23^. *Stat3*^*fl/fl*^ *Zic1-cre* mice displayed decreased vertebral body bone mass, and only a low rate of paraplegia (10%) (Fig 4. f, g). Conversely, deletion of *Schnurri3* (*Shn3; Hivep3*), a cell-intrinsic inhibitor of osteoblast function and bone formation^24,25^, with *Zic1-cre* led to an increase in bone mass within the vertebral body but no significant change in long bone parameters (Fig. 4h, i). Thus, *Zic1*-lineage vSSCs play an essential and specific role in producing the osteoblasts that form the vertebrae.

Next, we identified the human counterparts of murine vSSCs. First, we identified a population in the human endplate co-expressing ZIC1 and PAX1 (Fig. 4j). Flow cytometry also identified a population expressing a similar panel of markers as murine vSSCs (Lin-THY1-CD105-CD200+EMB-, hereafter human vSSCs) (Fig. 4k). Human vSSCs show similar stemness features as murine vSSCs, having the ability to form bone organoids after xenotransplantation into highly immunodeficient *NOD-scid* gamma (NSG) mice, including giving rise to chondrocytes and bone matrix forming osteoblasts (Fig. 4l, m). After transplantation, human vSSCs maintained themselves and concomitantly differentiated to other populations corresponding to the other cell types observed in the murine Zic1 vSSC-lineage (Fig. 4n). Thus, humans and mice share an analogous vSSC population.

The finding that a distinct vSSC is responsible for forming the vertebrae raises the possibility that many signature disease processes preferentially involving the spine are attributable to this vSSC. With this in mind, we were intrigued by longstanding unexplained clinical observations that a wide range of solid tumors, including breast, lung and prostate, display a marked metastatic preference for the spine over other skeletal sites, with up to five vertebral metastases for every one long bone metastasis in some contexts^26^. To evaluate the role of vSSCs in vertebral metastatic tropism, we first confirmed that preferential vertebral versus long bone tropism is conserved in mice using the caudal artery injection metastasis model^27^ with several syngeneic breast cancer cell lines, including E0771, 4T1.2 and Py8119 cells (Fig. 5a–d, Extended Data Fig. 9a, b). In line with the increased ultimate outgrowth of vertebral metastases seen, a greater number of initially seeding tumor cells were present at vertebrae than long bones (Fig. 5e–g). Interestingly, the site of initial seeding was predominantly in the marrow space in the primary spongiosum adjacent to the growth plate in long bones and the endplate in vertebrae (Fig. 5h), placing the initially seeding tumor cells in relative physical proximity to vSSCs and their immediate derivates. We next investigated whether passive alterations in blood flow contribute to the observed differences in tumor cell early seeding. Measurement of relative blood flow rates using fluorescent microspheres identified increased blood flow in long bones versus vertebrae (Extended Data Fig. 9c–e). Thus, the increased early tumor seeding of vertebrae cannot be explained by passive blood flow distribution. To identify whether vSSCs provide a basis for the high rates of vertebral versus long bone metastases, we examined the ability of bone organoids derived from *Zic1*-lineage vSSCs versus lbSSCs to recruit tumor cells in an *in vivo* competitive seeding assay. vSSCs and lbSSCs were isolated by FACS and transplanted into the thigh musculature in contralateral legs in the same host. After allowing these bone organoids of defined cellular composition to mature and mineralize for 4 weeks, host mice were challenged with Py8119 or 4T1.2 tumor cells by caudal artery injection (Fig. 5i, Extended Data Fig. 9g, j). Histology demonstrated that tumor cells infiltrated into these bone organoids and were physically adjacent to graft-derived skeletal cells (Fig. 5j, Extended Data Fig. 9m). Even after normalizing the surrounding anatomic context using this organoid system, *Zic1*-lineage vSSC-derived bone organoids displayed a higher rate of metastatic recruitment than that of lbSSCs (Fig. 5k, l, Extended Data Fig. 9h–l). In addition to showing that vSSCs are sufficient to drive increased metastatic seeding using organoids, we sought to establish that vSSCs are necessary for the high rates of metastatic seeding in the native vertebral microenvironment. *Zic1-cre Stat3*^*fl/fl*^ mice were used to attenuate the generation of *Zic1*-lineage vSSC-derived osteoblasts. *Zic1-cre Stat3*^*fl/fl*^ mice showed a decreased vertebral metastasis rate after caudal artery challenge with Py8119 cells, indicating that vSSCs contribute to metastasis in the native vertebral microenvironment (Fig. 5m).

Based on these findings, we searched for a vSSC-derived mediator of vertebral metastatic tropism, ultimately identifying milk fat globule epidermal growth factor 8 (*Mfge8*). *Mfge8* encodes a secreted protein with higher levels of expression in vSSCs than lbSSCs (Extended Data Fig. 10a, b). MFGE8 was sufficient to induce tumor cell migration *in vitro,* with Py8119 cells showing a dose-dependent migration in response to recombinant MFGE8 in a transwell assay (Fig. 5n, o). *Mfge8* was also necessary for tumor cell migration in a co-culture assay. Vertebral bone marrow stromal cells induced a greater migration of Py8119 cells in a transwell assay than long bone marrow stromal cells, and this effect was *Mfge8* dependent, as the same vertebral and long bone populations from *Mfge8*^*−/−*^ mice no longer displayed a differential ability to induce migration (Fig. 5p, q). MFGE8 also contributed to vertebral metastasis *in vivo*, as *Mfge8*^*−/−*^ mice displayed markedly reduced rates of vertebral metastasis after caudal artery challenge with Py8119 cells (Fig. 5r, s). In line with *Mfge8* being also expressed in lbSSCs, albeit at a lower level than in vSSCs, a more modest decrease in long bone metastases was observed, and *Mfge8*-deficiency ablated the difference between vertebral and long bone metastasis rates. In line with this, the number of early seeding tumor cells in vertebrae also significantly dropped in *Mfge8*^*−/−*^ mice (Fig. 5t, u). *Mfge8*^*−/−*^ mice displayed no baseline bone phenotype at 8 weeks of age (Extended Data Fig. 10c). To demonstrate that the reduced vertebral metastasis phenotype of *Mfge8*^*−/−*^ mice reflected vSSC metastatic function, bone organoids were generated from vSSCs derived cells from *Mfge8*^*−/−*^ and WT control mice in contralateral thigh musculature within the same hosts before caudal artery challenge with Py8119 cells. In this setting, the *Mfge8*-deficient vSSC-derived organoids displayed a markedly decreased rate of metastatic recruitment (Fig. 5v, w). Thus, MFGE8 is a key vSSC-derived mediator of vertebral tropism.

Here we established the concept that both the initial formation of vertebral bone and its subsequent postnatal maintenance is achieved by a novel stem cell type that is distinct from all previously studied long bone stem cells in terms of function, location and transcriptional program in both mice and humans. We also fundamentally advance the definition of skeletal stem cells, by identifying a new marker (EMBIGIN) that marks non-stem populations within current-definition SSCs within long bones and vertebrae, thereby removing these contaminating mature cells from stem cell preparations and enhancing future skeletal stem cell studies in all anatomic sites. Finally, this study offers an explanation for longstanding clinical observations about the vertebral metastatic tropism of breast cancer, mapping this effect at least in part to the specific vSSCs identified here and identifying MFGE8 as a vSSC-derived mediator of this effect.

## Methods

### Mice.

Floxed *Osterix* (*Osx*^*fl/fl*^) mice^28^ were a gift from B. de Crombrugghe (University of Texas MD Anderson Cancer Center). *PthrP-mCherry* mice^2^ were a gift from Noriaki Ono (UT Health School of Dentistry). *Rosa26-tTA* mice^29^ were a gift from Luigi Puglielli (University of Wisconsin-Madison). *Rosa26*^confetti^ (Stock 017492), *NOD scid gamma* (*NSG*, Stock 005557), *NSG-EGFP* (Stock 021937), *Rosa26*^mT/mG^ (Stock 007676), *R26-M2rtTA* (Stock 006965), *pTRE-H2BGFP* (Stock 005104), *Prrx1-cre* (Stock 005584), *Ocn-cre* (Stock 019509), *Pax7-cre* (Stock 010530), *Shh-cre* (Stock 005622), *Mpz-cre* (Stock 017927), *MIP-GFP* (*Ins1-EGFP*, Stock #: 006864), floxed *Stat3* (*Stat3*^fl/fl^) mice (Stock 016923) and *UBC-GFP* (Stock 004353) mice were purchased from Jackson Laboratories. *Mfge8*^*−/−*^ (RBRC01726) mice^30^ were purchased from RIKEN bioresource research center. *Shn3 loxp* mice^31^ were reported previously. All mice were maintained on a C57BL6/J background throughout the study except for experiments involving 4T1.2 cells that were conducted in BALB/cJ mice. All animals were maintained in accordance with the NIH Guide for the Care and Use of Laboratory Animals and were handled according to protocols approved by the Weill Cornell Medical College institutional animal care and use committee (IACUC).

### Cell lines.

Murine breast cancer cell lines 4T1.2 (CRL-3406^™^) and PY8119 (CRL-3278^™^) were purchased from ATCC. E0771(94A001) cells were purchased from CH3 Biosystems. All cell lines were stably transduced with Luciferase (selected by blastomycin) and EGFP (selected by puromycin) using lentiviral transduction to generate fluorescence/bioluminescence dual reporters.

### Generation of *Zic1-cre* and *Pax1-creER*^*T2*^ mice.

The *Zic1-cre* and *Pax1-creER*^*T2*^ knock-in mice were generated at Cyagen and on a C57/BL6 background (Extended Data Fig. 4). Briefly, the gRNA to mouse *Zic1* or *Pax1* gene, the donor vector containing “Cre-rBG pA” cassette or “*CreER*^*T2*^-rBG pA” cassette, and Cas9 mRNA were co-injected into fertilized mouse eggs to generate targeted conditional knockin offspring. F0 founder animals were identified by PCR followed by sequence analysis, which were bred to wildtype mice to test germline transmission and F1 animal generation. The *Zic1* gRNA target sequence was: gRNA1 (forward strand), CCGTGCAGCCACGATGCTCCTGG, gRNA2 (reverse strand), TCCGGCGTCCAGGAGCATCGTGG. The *Pax1* gRNA target sequence was: gRNA (reverse strand): AAGGTGAACTTCATCCGATTGGG.

### Tamoxifen injection.

**Fig. 2|:**
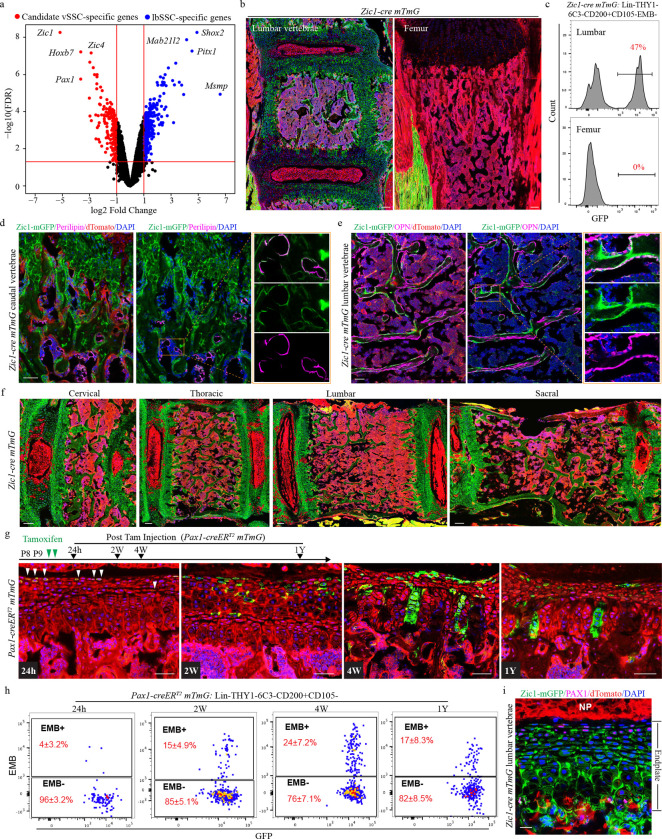
*Zic1-cre* and *Pax1-creER*^*T2*^ label candidate vSSCs. **a,** Volcano plot highlighting the differentially expressed transcriptional factors in candidate vSSCs and lbSSCs (FDR<0.05). **b,** Representative fluorescence images of the lumbar vertebra (L3, left) and femur (right) from *Zic1-cre mTmG* mice at P10. Scale bar, 100 μm. Data are representative of three independent experiments. **c,** Flow cytometry analysis of the mGFP+ *Zic1*-lineage cells within immunophenotypic SSCs (Lin-THY1-6C3-CD200+CD105-EMB- populations) in lumbar vertebrae (L1-L6) and femurs. n=4. **d,** Immunofluorescence staining of *Zic1-cre mTmG* mouse caudal vertebrae for Perilipin, showing the contribution of *Zic1*-lineage cells to marrow adipocytes. Data are representative of two independent experiments. Scale bar, 50 μm. **e,** Immunofluorescence staining of *Zic1-cre* mTmG mouse lumbar vertebrae with anti-OPN antibody, showing the contribution of *Zic1-cre* cells to osteoblasts. Data are representative of two independent experiments. Scale bar, 50 μm. **f,** Representative fluorescence images of the cervical, thoracic, lumbar and sacral vertebrae from *Zic1-cre mTmG* mice at 6-weeks of age. n=2 biological replicates. Scale bar, 100 μm. **g,** Tracing of *Pax1*-lineage cells in the endplate region of *Pax1-creER*^*T2*^ *mTmG* mice after a tamoxifen pulse on P8 and P9. n=4 biological replicates. Scale bar, 50 μm. **h,** Flow cytometry of *Pax1-creER*^*T2*^ *mTmG* mice 24h (n=6), 2w (n=8), 4w (n=10), or 1 year (n=6) after a tamoxifen pulse. Data are mean±s.d. **i,** Immunofluorescence staining of P4 *Zic1-cre mTmG* mouse lumbar vertebrae for PAX1, showing PAX1 expression in *Zic1*-lineage cells at the resting zone of vertebral endplate. Data are representative of two independent experiments. Scale bar, 20 μm.

Tamoxifen (Sigma, cat. T5648) was prepared in corn oil at 10 mg/ml. For lineage tracing analysis in Fig. 2g, h, Extended Data Fig. 5b, c, *Pax1-CreER*^*T2*^ *mTmG* mice received 800ug of tamoxifen intraperitoneally for two consecutive days at P8 and P9. For the *in vivo* clonality assay, *Pax1-CreER*^*T2*^ *Rosa26*^confetti^ mice received 1.5mg of tamoxifen intraperitoneally for six consecutive days at 1 month of age.

### Histone H2B-GFP label retention studies.

*pTRE-H2BGFP* mice were previously described^32^. To label slow-cycling cells under the control of a tetracycline response element, *pTRE-H2BGFP* mice were crossed with *ROSA:LNL:tTA* (tet-off) or *R26-M2rtTA* (tet-on) mice. For the tet-off system, 8-week-old mice were fed a doxycycline containing diet (200 mg/kg; Bio-Serv, cat. S3888) during a 6-month chase period. For tet-on system, 4-week-old mice were fed a doxycycline diet for 5 weeks followed by a chow diet and chase for 2 month or 14 months as indicated in Extended Data Fig. 6c.

### Cell Isolation for FACS.

Mouse vertebrae (vertebral bodies from the cervical to sacral region) and long bones (tibia and femur) tissues or human endplate samples were minced by razor blades followed by the first round of enzymatic digestion with Collagenase P (1 mg/ml; Roche, cat. 11213857001) and Dispase II (2 mg/ml; Roche, cat.04942078001) in αMEM medium containing 2% FBS at 37 °C with agitation for 20min. DNase I was then added directly to the digestion buffer at 10 units/ml and tissue was incubated at 37 ° for 10min. The supernatant was transferred to a 50 ml tube with 30ml cold αMEM medium(2% FBS) and the remaining tissue was subjected to the second round of digestion for 30min with fresh digestion buffer (1 mg/ml Collagenase P, 2mg/ml Dispase II and 2 units/ml DNase I). The supernatants from both rounds of digestion were combined and filtered through a 70-μm nylon mesh. Tubes were centrifuged and the resulting cell pellet was subjected to FACS.

### Fluorescence-activated cell sorting (FACS).

Prepared single-cell suspensions were washed twice with ice-cold FACS buffer (2% serum + 1 mM EDTA in PBS) and incubated with Fc blocking buffer (1:100 dilution; BD bioscience, cat. 553142 for mouse and cat. 564765 for human) for 15 min at 4 °C. Primary antibody dilutions were prepared in Brilliant Stain Buffer (BD bioscience, cat. 563794). Cells were incubated in the dark for 30 min at 4 °C with primary antibody solution and washed 2 times with FACS buffer. When secondary antibodies were used, cells were further incubated with secondary antibody solution for 20 min. Cells were then washed two times and re-suspended in FACS buffer with DAPI (BD bioscience, cat. 564907). FACS was performed using a Becton Dickinson Aria II equipped with 5 lasers (BD bioscience). Beads (Invitrogen, cat. 01-3333-42) were used to set initial compensation. Fluorescence minus one (FMO) controls were used for additional compensation and to assess background levels for each stain. Gates were drawn as determined by internal FMO controls to separate positive and negative populations for each cell surface marker. Typically, 2.5 million events were recorded for each FACS analysis, and the data were analyzed using FlowJo (v10.8.1).

### FACS antibodies.

Antibodies for FACS of murine samples included CD31 (MEC13.3, BD Bioscience), CD45 (clone 30-F11, BD Bioscience), Ter119 (clone TER-119, BD Bioscience), CD249/BP-1 (clone 6C3, eBioscience), THY1.2 (clone 53-2.1, BD Bioscience), CD200 (clone OX-90, BD Bioscience), CD105 (clone MJ7/18, BioLegend), EMB (clone REA501, Miltenyi Biotec). Antibodies for FACS of human samples included CD31 (clone WM59, BioLegend), CD45 (clone HI30, BioLegend), CD235a (clone GA-R2, BioLegend), THY1-1 (clone 5E10, BD Bioscience), CD200 (clone MRC OX-104, BD Bioscience), CD105 (clone 43A3, BioLegend), EMB (Rabbit monoclonal [EPR11417], Abcam), CD146 (clone P1H12, BD Bioscience), Podoplanin (clone LpMab-17, BD Bioscience), CD73 (clone AD2, BD Bioscience), CD164 (clone N6B6, BD Bioscience), HLA-ABC (clone W6/32, BioLegend), B2M(beta2-microglobulin) (Clone 2M2, BioLegend), Goat anti-Rabbit IgG Alexa Fluor Plus 647 (polyclonal, A32733, Invitrogen) and Donkey anti-Rabbit IgG Alexa Fluor Plus 555(polyclonal,A32794, Invitrogen).

### μCT scans and analysis.

μCT analysis was conducted on a Scanco Medical μCT 35 system at the Citigroup Biomedical Imaging Core as previously described^33^. Briefly, a Scanco Medical μCT 35 system with an isotropic voxel size of 12 μm was used to image the distal femur and vertebrae. Scans were conducted in PBS and used an X-ray tube potential of 55 kVp, an X-ray intensity of 0.145 mA, and an integration time of 600 ms. μCT analysis was performed by an investigator blinded to the genotypes of the animals.

### Intramuscular transplant for bone organoids formation and differentiation hierarchy.

Intramuscular transplantation was performed in 6- to 8-week-old *MIP-GFP* hosts for transplantation of mouse cells or *NSG/NSG-EGFP* mice for transplantation of human cells. A 1 mm longitudinal incision was made on the right hindlimb, and the right anterolateral femur was exposed. The vastus lateralis muscle was dissected and a 2–3 mm muscle pouch was surgically created. A surgifoam absorbable gelatin sponge (Ethicon, cat. 1972) containing sorted cell populations of interest was placed into the muscle pouch. The overlying fascia was closed by using 4–0 polyglactin 910 absorbable sutures (Ethicon, cat. J386), and wound clips were used to close the skin incision. For bone organoid formation, 20,000–50,000 of the indicated cells were transplanted. Mouse bone organoids at different stages were harvested after 2 weeks, 6 weeks or 4 months post-surgery, and human bone organoids were harvested after 6 weeks post-surgery. For murine cells serial transplantation studies, 100,000 of the indicated mouse cells were used for the first round of transplantation. For differentiation hierarchy studies on human cells, human vSSCs were sorted by FACS from an endplate specimen and expanded *in vitro* in human MSC Basal Medium (Cat #05401, Stem cell technologies) with 10% Human Platelet Lysate (Cat # 06960, Stem cell technologies) and Fibronectin (1:1k, Cat # 07159, Stem cell technologies), 100,000 vSSCs were then labeled with DiD lipophilic dye-647 (Cat#V22887) and transplanted into murine *NSG-EGFP* hosts. Animals were euthanized by CO_2_ narcosis 7–10 days post-surgery, and the muscle was dissociated for FACS analysis.

### Mammary fat pad transplantation.

Mammary fat pad transplantation was performed in 6- to 8-week-old *MIP-GFP* hosts as previously described^1^. Briefly, 30,000 EMB− and EMB+ candidate vSSCs (Lin-THY1-6C3-CD200+CD105-GFP+) were isolated by FACS from vertebrae of *UBC*-*GFP* mice at P4. Cells were then transferred into a surgifoam absorbable gelatin sponge and placed in the inguinal mammary gland. Animals were euthanized by CO_2_ narcosis 7 days post-surgery, and the mammary fat pad was dissociated for FACS analysis.

### Sample preparation for cryo-sectioning and imaging.

Freshly extracted mouse samples were fixed with 4% PFA overnight at 4 °C. Samples were washed with PBS and decalcified with daily changes of 0.5 M EDTA for 1–5 days depending on the age of the samples. Samples were incubated with infiltration medium (20% sucrose + 2% polyvinylpyrrolidone in PBS) with rocking until they sank to the bottom of the tube. Embedding was performed with embedding medium (OCT + 15% sucrose in PBS) and samples were preserved at −80 °C. Sections, 10–20 μm in thickness, were cut using a Leica cryostat.

### Paraffin embedding:

Specimens were fixed overnight with 4% PFA at 4°C. Samples were decalcified with 0.5 M EDTA for 5–6 days and then subjected to paraffin embedding. The Center for Translational Pathology at Weill Cornell performed paraffin embedding, sectioning and staining (hematoxylin and eosin) following a previously published protocol^34^.

### Whole-mount skeletal staining.

Mice were eviscerated, and the skin was removed, and the resulting samples were transferred into acetone for 48 hours after overnight fixation in 95% ethanol. Skeletons were then stained in Alcian blue and Alizarin red solution as described previously^35^. Specimens were kept in 1% KOH until tissue had completely cleared.

### Bone blood flow measurements.

WT C57BL6/J mice were given caudal artery injection of 300μl fluorescent microspheres (Invitrogen FluoSpheres 15 μm, cat. no. F21010). Mice were killed 1 min after injection and vertebrae and long bone were collected immediately for FACS analysis.

### Cancer cell line injection.

Caudal artery injection was performed as described previously^27^. For the bone metastasis model, PY8119/luc-GFP (3X10^5^ cells to C57B/6 female), 4T1.2/luc-GFP (5X10^5^ cells to BALB/c female), E0771/luc-GFP(5X10^5^ cells to C57B/6 female) suspended in 200 μl PBS were injected through caudal artery using a 30G syringe needle. For bone organoids metastasis model, PY8119/luc-GFP (1X10^6^ cells to C57B/6 female), 4T1.2/luc-GFP (1X10^6^ cells to BALB/c female) were injected through the caudal artery. For early seeding analysis in vertebrae and long bones, PY8119/luc-GFP (5X10^5^ cells to C57B/6 female) were injected via the caudal artery. For early seeding analysis in bone organoids, 4T1.2/luc-GFP (1X10^6^ cells into BALB/c female) cells were injected via the caudal artery.

### Transwell migration assay.

5.0 μm Pore Polycarbonate Membrane Inserts(3421,Corning) were placed into the wells of a 24-well plate and 600 μl serum-free F-12K medium containing 0, 100, 1000ng/ml recombinant MFGE8 (10853-H08B, Sino Biological Inc.) was added to the outer compartment of each well. A 400μL suspension of 1X10^5^ PY8119 cells/ml in serum-free F-12K medium was gently added onto the cell culture inserts. Cells were then incubated at 37 °C for 18h. For bone cell and tumor cell chemotaxis experiments, 2.5X10^5^ bone marrow stromal cells from mouse vertebrae and long bones were seeded at the bottom of the wells in serum-free αMEM medium, and a 400μL suspension of 1X10^5^ PY8119 cells/ml in serum-free αMEM medium was gently added onto the cell culture inserts. Cells were then incubated at 37 °C for 18h. Cells that did not undergo migration on the top of membrane were carefully removed with cotton swabs. Migrated cells on the inserts were fixed in 4% paraformaldehyde for 10min and washed with PBS 3 times followed by staining with 0.5% crystal violet solution in 25% methanol at room temperature for 10 minutes. Cells were then washed 4 times in water and dried at room temperature.

### Bulk RNA sequencing.

Total RNA was freshly extracted from FACS-isolated populations at P10 WT mice using the RNeasy Plus Micro Kit (Qiagen, cat. 74034). RNA quality and quantity were verified by using an Agilent 2100 Bioanalyzer system. cDNA libraries were generated using the Illumina SMART-Seq v4 plus Nextera XT DNA Library Preparation Kit and sequenced on a NovaSeq 6000 sequencer. Differential gene expression analysis was performed using the DESeq2 package. Only false discovery rate (FDR)-adjusted *P* values of less than 0.05 were considered statistically significant. Normalized read counts were used for generating heatmap plots. The data in Extended Data Fig 2a was visualized by heatmap with hierarchical clustering in R v3.6.2 with the pheatmap package v1.0.12. Heatmaps in Extended Data Figure 3c and Extended Data Figure 10a were generated by Heml 1.0^36^.

### Human endplate samples collection.

The study was approved by the Hospital for Special Surgery institutional review board (2021-0408). Patients between the ages of 18–85 were enrolled if they were undergoing cervical decompression and fusion, anterior lumbar interbody fusion, lumbar transforaminal interbody fusion, or lumbar laminectomy. Patients were excluded if they were under the age of 18. Informed consent was obtained pre-operatively from all patients prior to enrollment. Endplate samples were collected between November 2021 to August 2022.

### Statistical analyses.

All data are shown as mean ± s.d. as indicated unless otherwise specified. For comparisons between two groups, unpaired, two-tailed Student’s *t* tests were used. For comparisons of three or more groups, one-way or two-way ANOVA was used if normality tests passed, followed by Tukey`s multiple comparison test for all pairs of groups. For comparisons of tumor metastasis rate between two groups, Chi-square test was used. GraphPad PRISM v7.04 was used for statistical analysis. P values are shown (*P < 0.05, **P < 0.01, ***P < 0.001, ****P < 0.0001, ns: not significant).

## Extended Data

**Extended Data Fig. 1 |:**
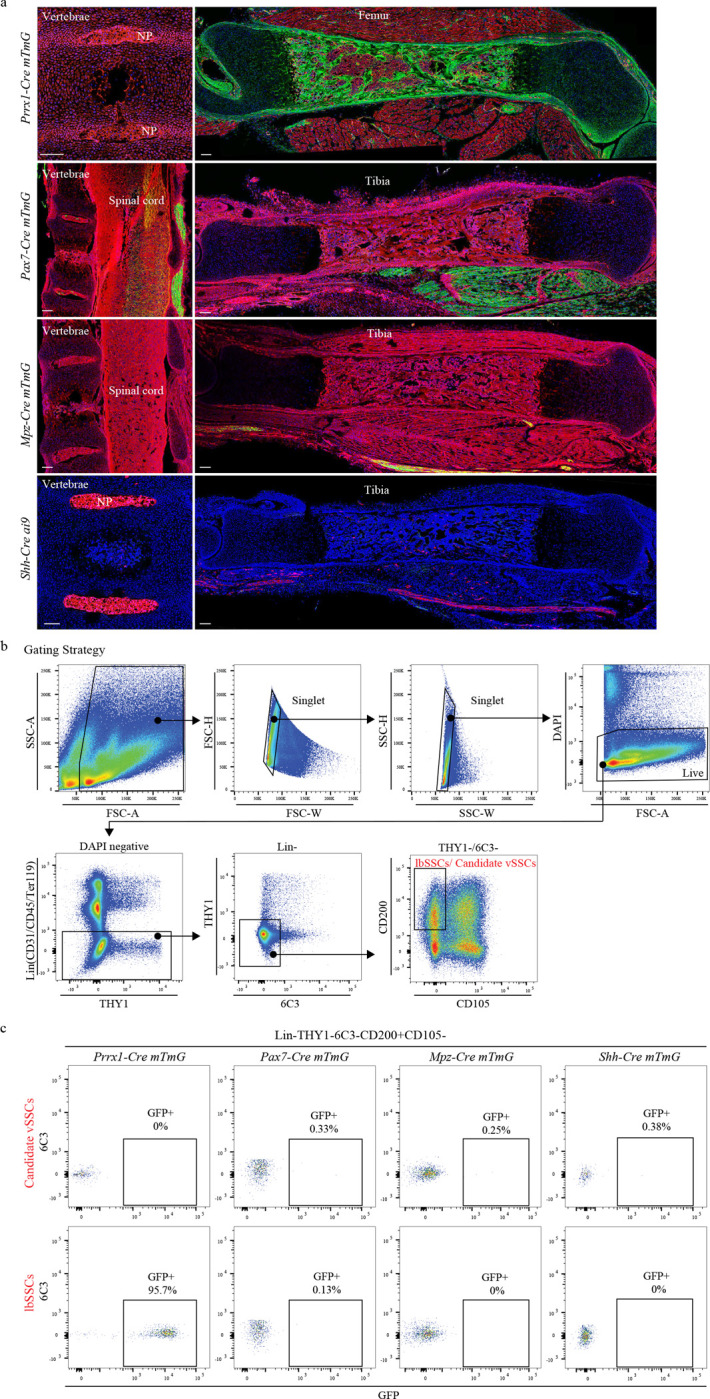
Exclusion of potential lineage marker for vertebrae. **a,** Representative fluorescence images of vertebrae and long bone from *Prrx1-cre mTmG, Pax7-cre mTmG, Mpz-cre mTmG* and *Shh-cre ai9* mice at P1. NP, nucleus pulposus. n=2 for each mouse line. Scale bar, 100 μm. **b,** Gating strategy for FACS of SSCs in long bones (lbSSCs) and candidate SSCs in vertebrae (candidate vSSCs). **c,** Flow cytometry for the contribution of different cre lineage cells to lbSSCs or candidate vSSCs. n=2 or 3 for each mouse line.

**Extended Data Fig. 2 |:**
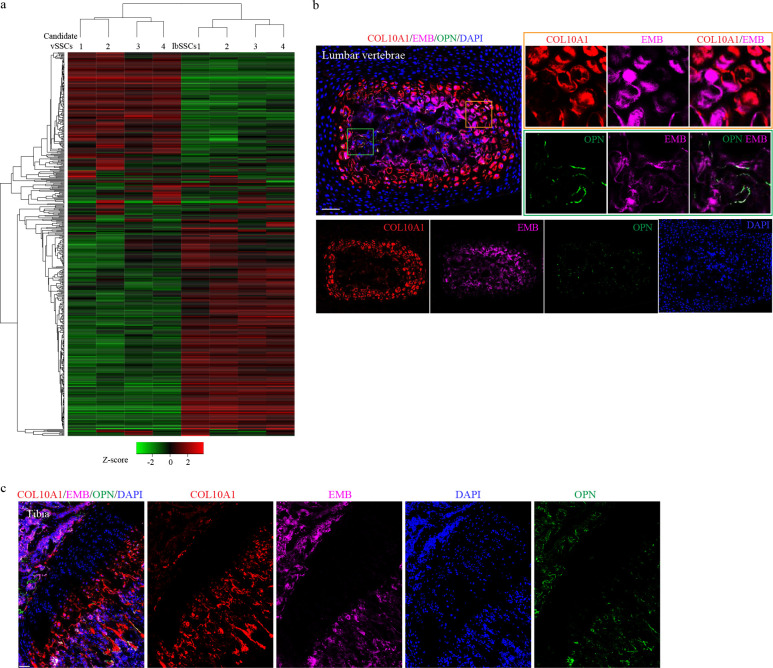
EMB labels a non-stem population **a,** Clustering heatmap showing the differentially expressed genes in candidate vSSCs (Lin-THY1-6C3-CD200+CD105-) versus lbSSCs (Lin-THY1-6C3-CD200+CD105-). Each row represents one gene, and each column represents one sample. **b&c,** Immunofluorescence staining for COL10A1, EMB and OPN on P10 mouse vertebrae (**b**) and tibia (**c**) sections, showing the expression of EMB in osteoblasts and hypertrophic chondrocytes. Images are representative of two independent experiments. Scale bar, 50 μm.

**Extended Data Fig. 3 |:**
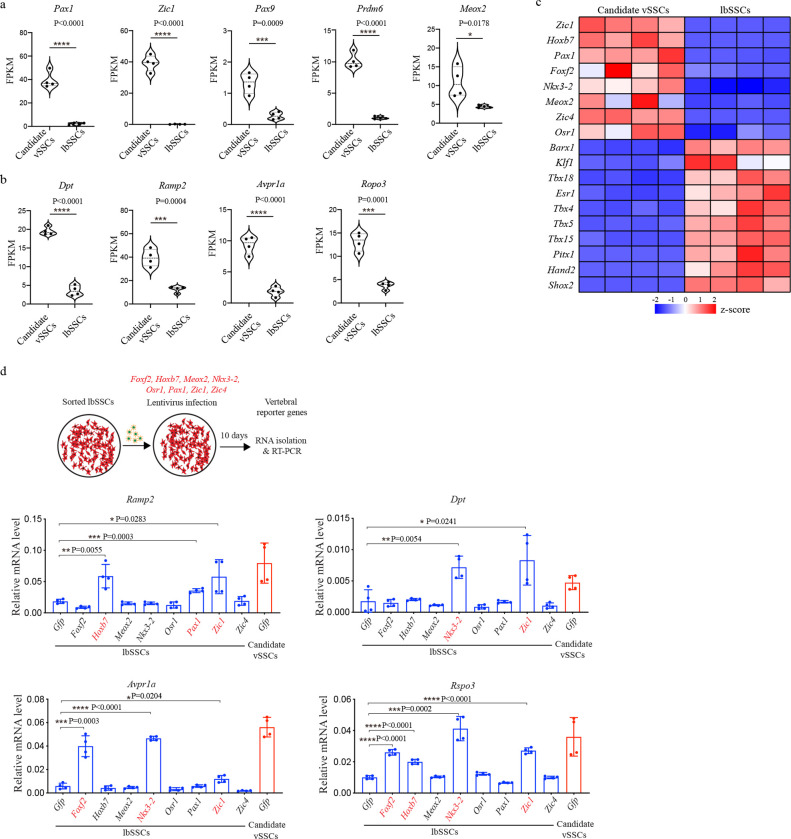
Identification of vSSC markers. **a,** Violin plot showing the expression of *Pax1, Zic1, Pax9, Prdm6* and *Meox2* genes in candidate vSSCs and lbSSCs as determined by RNA-seq. n=4, data are presented as mean±s.d., unpaired, two-tailed Student’s t test. **b,** Violin plot showing the expression of *Dpt, Ramp2, Avpr1a* and *Ropo3* genes in candidate vSSCs and lbSSCs as determined by RNA-seq. n=4, data are presented as mean±s.d., unpaired, two-tailed Student’s t test. **c,** Heatmap showing the top differentially expressed transcription factors in candidate vSSCs and lbSSCs. **d,** Diagram of the screen of vertebral-specific transcription factors in lbSSCs (top) and RT-PCR showing the activation of vertebral reporter genes in lbSSCs overexpressing single vertebral-specific transcription factors. n=4, data are presented as mean±s.d., unpaired, two-tailed Student’s t test.

**Extended Data Fig. 4 |:**
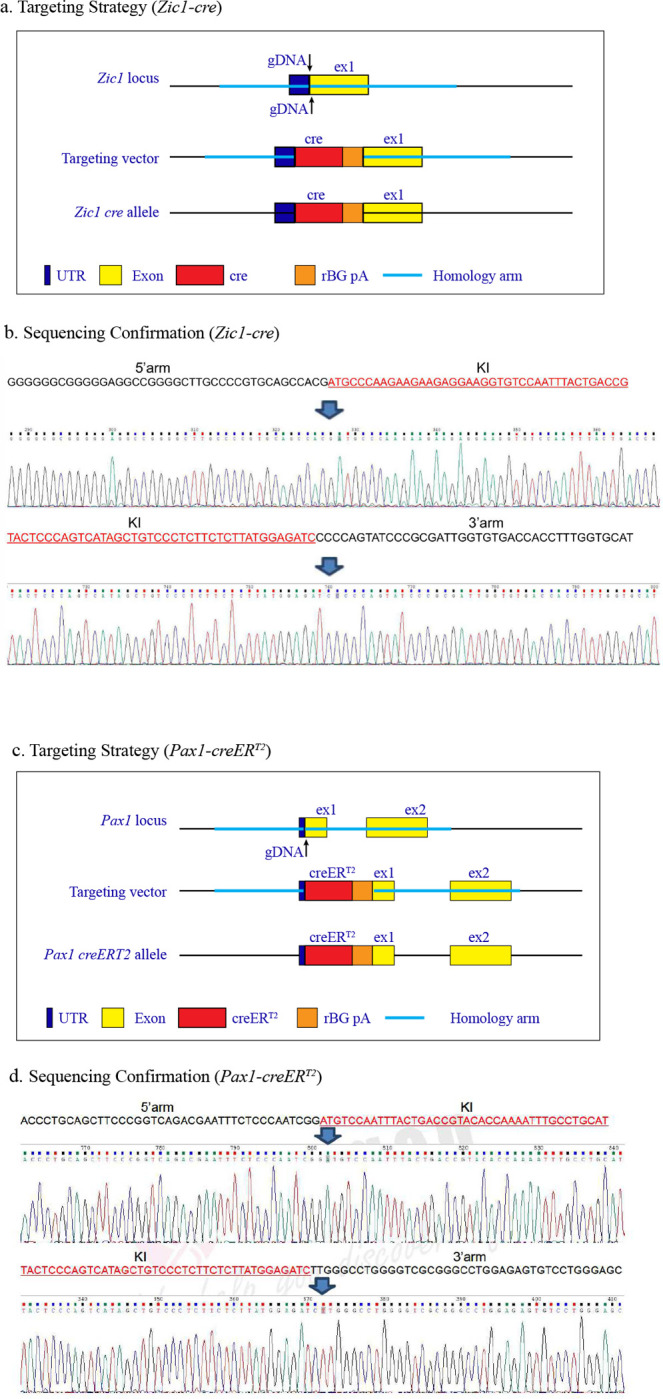
Generation of *Zic1-cre* and *Pax1-creER*^*T2*^ knock-in mouse lines. **a,** Generation of *Zic1-cre* line using CRISPR-Cas9. Structure of *Zic1* locus (top), targeting vector (middle) and knock-in allele (bottom). **b,** DNA sequencing result of the knock-in region. The sequence of knock-in element is labeled as red. **c,** Generation of *Pax1-creER*^*T2*^ line using CRISPR-Cas9. Structure of *Pax1* locus (top), targeting vector (middle) and knock-in allele (bottom). **d,** DNA sequencing result of the knock-in region. The sequence of knock-in element is labeled as red.

**Extended Data Fig. 5 |:**
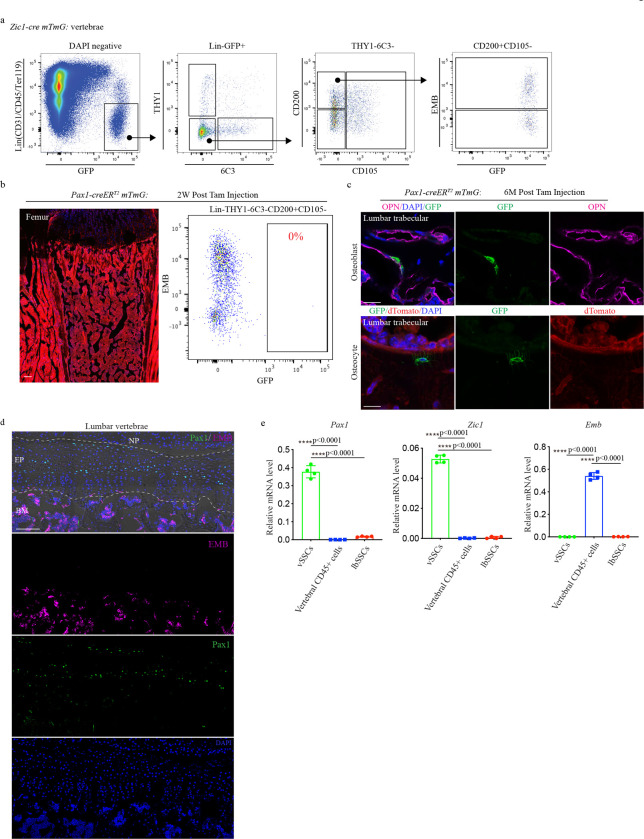
*Zic1-cre* and *Pax1-creER*^*T2*^ label vertebrae. **a,** Flow cytometry analysis of *Zic1-cre* cells with *mTmG* reporter in vertebrae at P10. n=5. **b,** Representative fluorescent image (left) and flow cytometry analysis (right) of femur from *Pax1-creER*^*T2*^ *mTmG* mice 2 weeks after Tamoxifen injections at P8 and P9. Data are representative of three independent experiments. Scale bars, 100 μm. **c,** Immunofluorescence images of 6-month-old *Pax1-creER*^*T2*^ *mTmG* mouse lumbar vertebral section (Tamoxifen induction at P8&P9), showing the contribution of *Pax1-creER*^*T2*^ cells to osteoblasts with anti-OPN antibody staining (top), and osteocyte in trabecular bone. Data are representative of three independent experiments. Scale bars, 20 μm. **d,** Immunofluorescence staining for EMB and PAX1 on 4-week-old mouse vertebrae sections. NP, nucleus pulposus, EP, endplate, BM, bone marrow. Data are representative of two independent experiments. Scale bars, 100 μm. **e,** RT-PCR analysis of *Pax1*, *Zic1*, and *Emb* genes in FACS sorted *Zic1*-lineage vSSCs, lbSSCs and vertebral CD45+ cells. n=4, data are presented as mean±s.d., one-way ANOVA followed by Tukey’s multiple comparison test.

**Extended Data Fig. 6 |:**
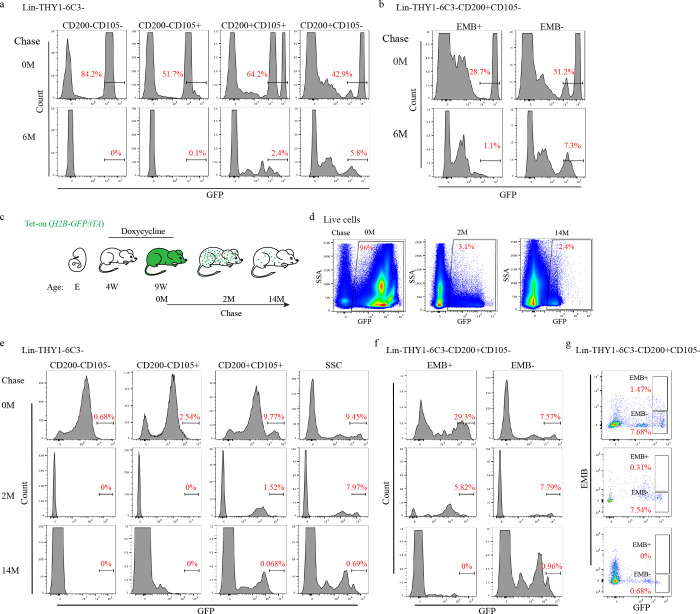
vSSCs are label retaining cells. **a&b,** Flow cytometry of H2B-GFP^hi^ label retaining cells in the vertebrae of *H2B-GFP/rtTA* mice before and after 6 months of doxycycline treatment. **c,** Diagram of label retention experiment: doxycycline food was administrated to the *H2B-GFP/tTA* mice to turn on GFP expression from 4-week-old to 9-week-old and replaced with chow diet after 9-week-old to turn off GFP expression, label retaining cells were analyzed 2 months or 14 months after doxycycline treatment. **d,** Representative flow cytometry plots showing the total GFP+ cells in vertebrae of *H2B-GFP/tTA* mice at indicated time points. **e-g,** Flow cytometry analysis of H2B-GFP^hi^ label retaining cells in vertebrae of *H2B-GFP/tTA* mice before and after 2-month or 14-month doxycycline treatment.

**Extended Data Fig. 7 |:**
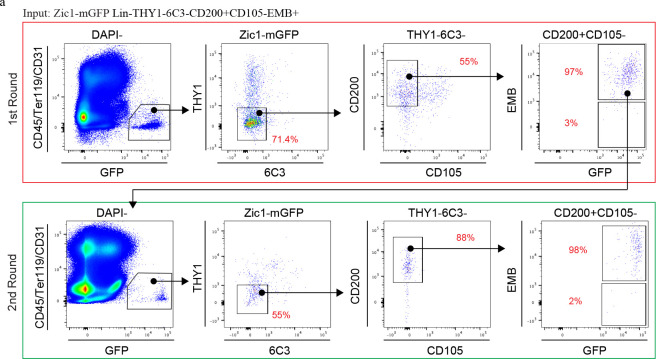
*Zic1*-lineage EMB+ cells lack stem cell properties. **a,** Flow cytometry of the cell populations derived from *Zic1*-lineage/Lin-THY1-6C3-CD200+CD105-EMB+ cells after the first round (top panels) and second round (bottom panels) of intramuscular transplantation. Plots are representative of 3 independent experiments.

**Extended Data Fig. 8 |:**
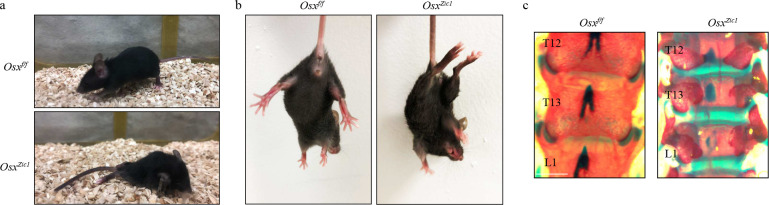
Deletion of key osteoblast factors in *Zic1-cre* cells lead to vertebral mineralization defects and hindlimb paralysis. **a,** Representative images showing the hindlimb paralysis phenotype observed in 4-week-old *Osx*^*zic1*^ mice. **b,** Splay reflex test showing that 4-week-old *Osx*^*f/f*^ mice have a normal limb splaying reflex while *Osx*^*zic1*^ mice display spasticity due to paraplegia. **c,** Whole-mount skeletal staining of 4-week-old *Osx*^*zic1*^ and *Osx*^*f/f*^ mice at the thoracolumbar region showing the mineralization defect of the dorsal vertebrae in *Osx*^*zic1*^ mice. n=3.

**Extended Data Fig. 9 |:**
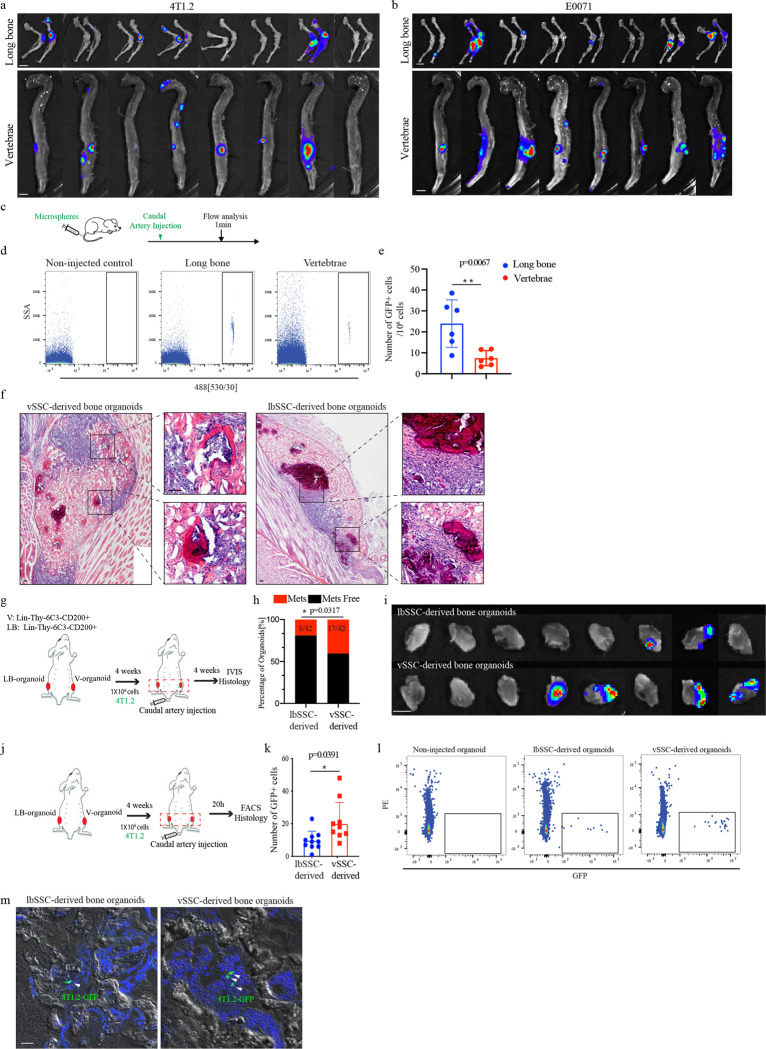
The tropism of breast cancer cells vertebrae. **a,** Representative bioluminescence images showing cancer metastasis in long bones and vertebrae 4 weeks after injection of 4T1.2 cells through the caudal artery. Scale bar, 5 mm. **b,** Representative bioluminescence images showing cancer metastasis in long bones and vertebrae 3 weeks after injection of E0771 cells through the caudal artery. **c**, Diagram of the blood flow distribution experiment. **d,** Representative flow cytometry plots showing the microspheres in long bone and vertebrae 1min after caudal artery injection. **e,** Quantification of the microspheres in long bone and vertebrae 1 min after caudal artery injection. n=6, data are presented as mean±s.d., unpaired, two-tailed Student’s t test. **f,** Representative histology images of H&E staining showing metastasized PY8119 cells in lbSSC-derived and vSSC-derived bone organoids 3 weeks after cancer cells injection, scale bar 200 μm. **g,** Diagram of the study of 4T1.2 metastasis to bone organoids. **h,** Quantification of the metastasis rate of 4T1.2 cells to lbSSC-derived and vSSC-derived bone organoids 3 weeks after cancer cells injection. n=42. Chi-square test. **i,** Representative bioluminescence images showing 4T1.2 metastasis in lbSSC-derived and vSSC-derived bone organoids 3 weeks after cancer cells injection. Scale bar, 5 mm. **j**, Diagram of the bone organoid early seeding experiment. **k,** Quantification of the early seeding cancer cells in lbSSC-derived and vSSC-derived bone organoids 20h after cancer cells injection. n=9, data are presented as mean±s.d., unpaired, two-tailed Student’s t test. **l.** Representative flow cytometry plots showing the early seeding cancer cells in lbSSC-derived and vSSC-derived bone organoids 20h after cancer cells injection. **m,** Representative histology images of three independent experiments showing the early seeding of cancer cells in lbSSC-derived and vSSC-derived bone organoids 20h after 4T1.2 cancer cells injection, scale bar, 20 μm.

**Extended Data Fig. 10 |:**
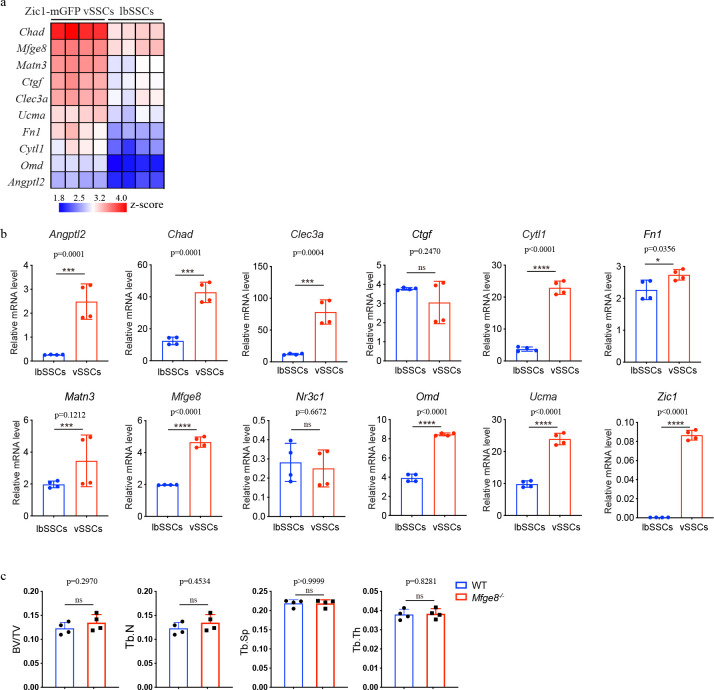
Identification of vSSC secreted proteins mediating breast cancer metastatic tropism. **a,** Heatmap showing the top differentially expressed secreted proteins in *Zic1-lineage* vSSCs versus lbSSCs. **b,** qPCR analysis of candidate vertebral-derived metastasis tropism factors in FACS isolated *Zic1*-lineage vSSCs (Lin-THY1-6C3-CD200+CD105-EMB-GFP+) and lbSSCs (Lin-THY1-6C3-CD200+CD105-EMB-). n=4, data are presented as mean±s.d., unpaired, two-tailed Student’s t test. **c,** Quantification of bone volume/total volume (BV/TV), trabecular number (Tb.N), trabecular thickness (Tb.Th) and trabecular separation (Tb.Sp) of L5 vertebrae in 8-week-old *Mfge8*^*−/−*^ and WT mice. n=4, data are presented as mean±s.d., unpaired, two-tailed Student’s t test.

## Figures and Tables

**Fig. 1 |:**
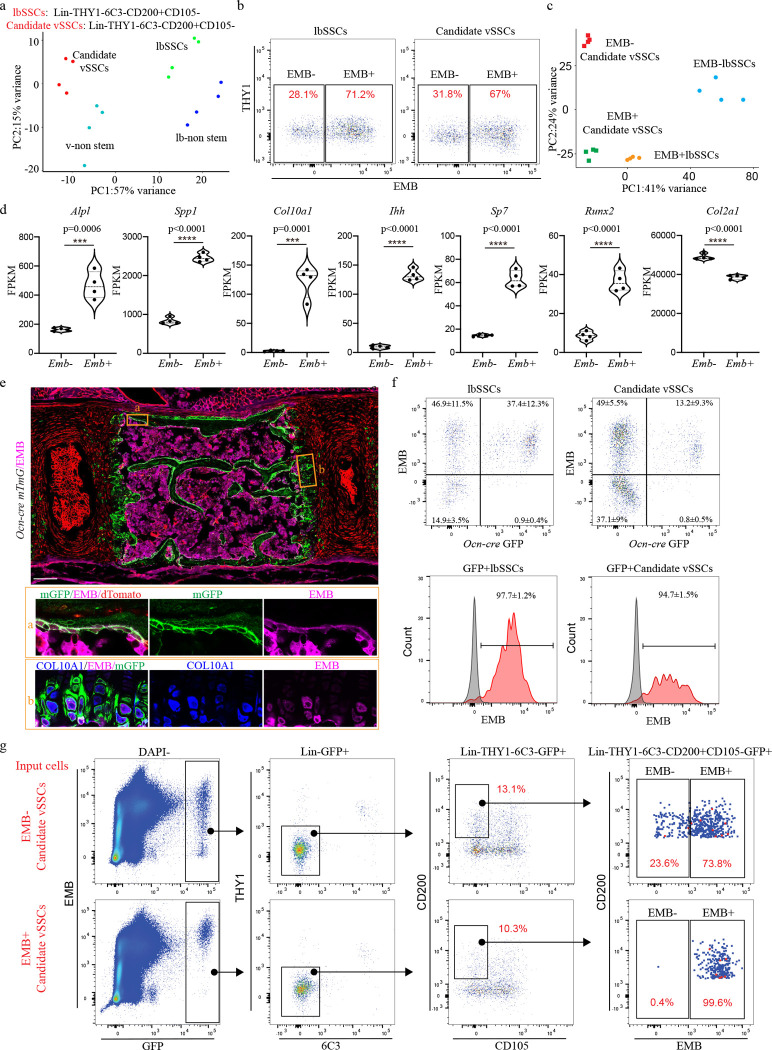
Identification of vSSC markers. **a**, Principal component analysis (PCA) of RNA-Seq following FACS of Lin-THY1-6C3-CD200+CD105- populations from the vertebrae (candidate vSSCs) and long bone(lbSSCs) of P10 mice. **b**, Representative flow cytometry analysis of the EMB+ and EMB− fraction of lbSSCs and candidate vSSCs. n=3. **c,** Principal component analysis (PCA) of RNA-seq following FACS of Lin-THY1-6C3-CD200+CD105-EMB+ and Lin-THY1-6C3-CD200+CD105-EMB- populations from the vertebrae and long bones of P10 mice. **d,** RNA-seq analysis using a Violin plot showing the expression of *Alpl, Spp1, Col10a1, Ihh, Sp7, Runx2* and *Col2a1* in EMB− candidate vSSCs and EMB+ candidate vSSCs. n=4, data are presented as mean±s.d., unpaired, two-tailed Student’s t test. **e,** Immunostaining for EMB and COL10A1 on thoracic vertebrae (T10) from *Ocn-cre mTmG* mice at 1-month of age. Data are representative of two independent experiments. Scale bar, 100 μm. **f,** Flow cytometry analysis of *Ocn-cre mTmG* vertebral cells. n=5 mice. Data are presented as mean±s.d. In the histogram pictures, grey peaks represent FMO control cells for EMB staining and red peaks represent GFP+lbSSCs or GFP+ Candidate vSSCs. **g,** Flow cytometry analysis of cells derived from EMB+ vs EMB− candidate vSSCs 7 days after mammary fat pad transplantation and organoid formation. Plots are representative of 3 independent experiments.

**Fig. 3 |:**
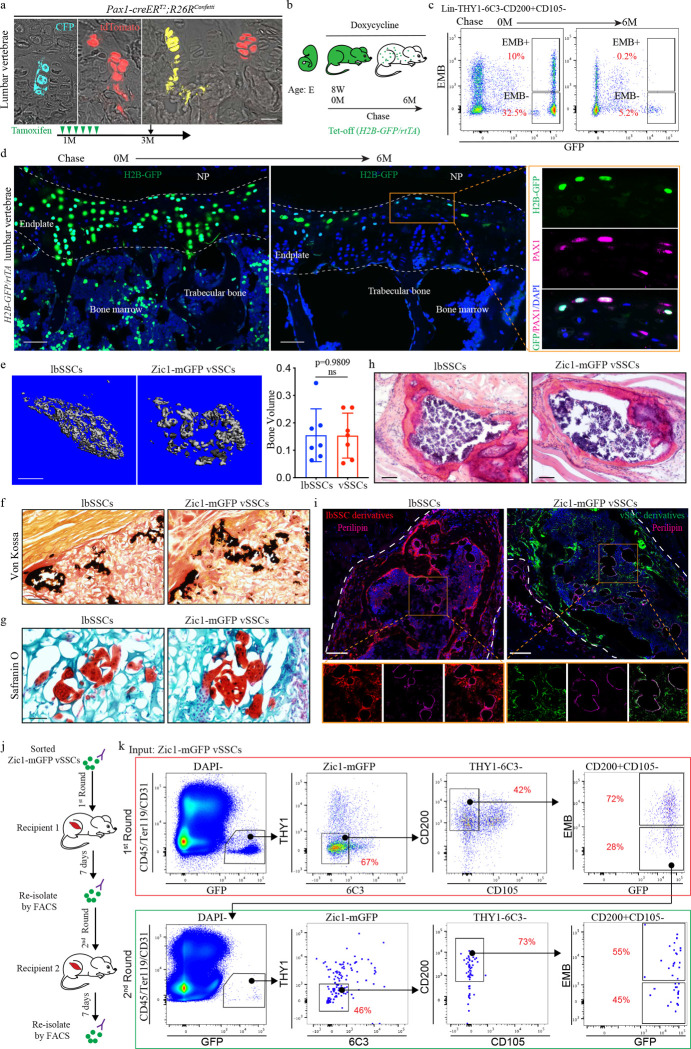
vSSCs fulfill stemness criteria. **a**, *In vivo* clonal analysis of *Pax1-creER*^*T2*^ cells at the vertebral endplate region in 3-month-old mice after tamoxifen induction at 1 month. Representative of 4 biologic replicates. Scale bar, 20 μm. **b**, Summary diagram for label retention studies: doxycycline chow was administrated to *H2B-GFP/rtTA* mice to suppress *de novo* H2B-GFP expression starting at 8-weeks of age. Label retaining cells were analyzed 6 months after initiating doxycycline treatment. **c**, Flow cytometry analysis of H2B-GFP^hi^ cells (label retaining cells) before and after doxycycline treatment. Representative of 4 biologic replicates. **d,** Immunostaining for PAX1 showing expression of PAX1(magenta) in H2B-GFP (green) label retaining cells at the vertebral endplate in 8-month-old *H2B-GFP/rtTA* mice, after 6 months of chase. Representative of 2 independent experiments. Scale bar, 50 μm. **e,** uCT analysis of lbSSC (Lin-THY1-6C3-CD200+CD105-EMB-) and *Zic1*-lineage vSSC-derived bone organoids 6 weeks after intramuscular transplantation showing 3D reconstruction (left) and quantification of bone volume (right). n=7 organoids/host mice per group. Data are mean±s.d., unpaired, two-tailed Student’s t test. Scale bar: 0.5 mm. **f,** Representative Von Kossa staining (black) for mineralized bone in lbSSC and *Zic1*-lineage vSSC-derived bone organoids 8 weeks after intramuscular transplantation. n=4. Scale bars, 500 μm. **g,** Representative images of Safranin O staining (red) for cartilage in lbSSC and *Zic1*-lineage vSSC-derived bone organoids 2 weeks after intramuscular transplantation. n=4. Scale bars, 200 μm. **h,** Representative images of H&E staining showing marrow recruitment in the bone organoids 4 month after intramuscular transplantation. n=3. Scale bars, 500 μm. **i,** Immunostaining of Perilipin+ adipocytes in lbSSCs and *Zic1*-lineage vSSC-derived bone organoids 4 months after intramuscular transplantation. n=3. Scale bars, 500 μm. **j,** Schematic diagram for the *in vivo* serial transplantation experiment. **k,** Flow cytometry analysis of vSSC-derived cell populations after the first round (top panels) and second round (bottom panels) of intramuscular transplantation. Plots are representative of 3 independent experiments.

**Fig. 4|:**
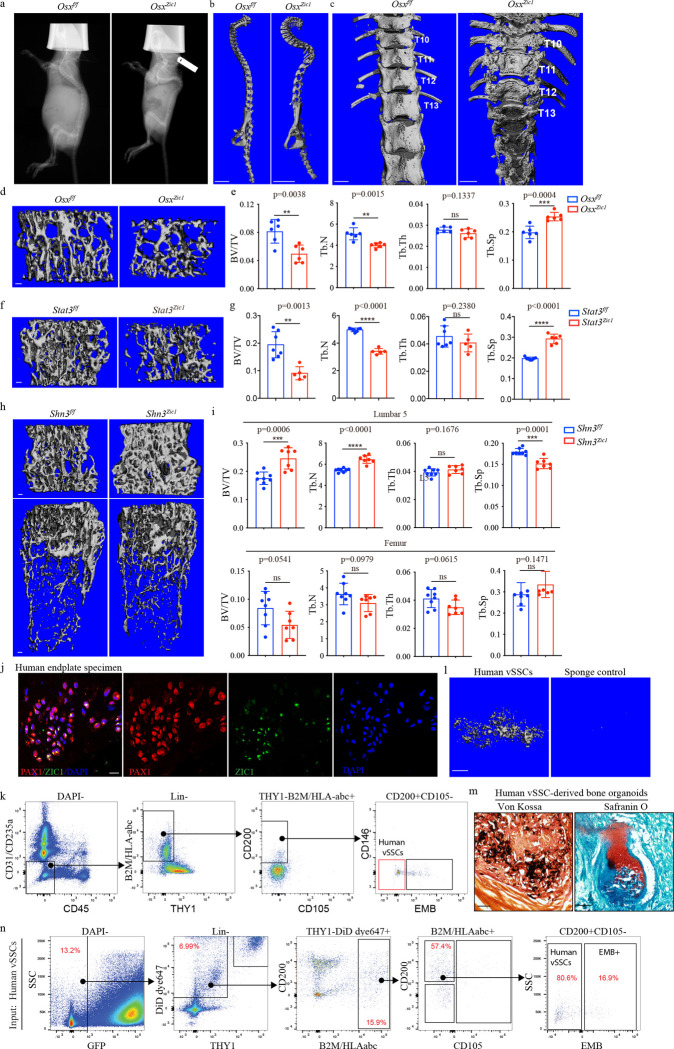
vSSCs contribute to physiologic mineralization. **a**, Representative X-ray images showing vertebral mineralization defects in 4-week-old *Osx*^*zic1*^ mice. n=2. **b**, 3D reconstruction of spine μCT images from 4-week-old *Osx*^*zic1*^ and *Osx*^*f/f*^ mice. Scale bar, 5 mm. **c**, Dorsal view of a 3D reconstruction of thoracic and lumbar spine μCT images showing the absence of the neural arch in 4-week-old *Osx*^*zic1*^ mice. Scale bar, 1 mm. **d,** 3D reconstruction of L5 vertebra μCT from 4-week-old *Osx*^*zic1*^ and *Osx*^*f/f*^ mice. Scale bar, 100 μm. **e,** Quantification of bone volume/total volume (BV/TV), trabecular number (Tb.N), trabecular thickness (Tb.Th) and trabecular separation (Tb.Sp) of L5 vertebra in 4-week-old *Osx*^*zic1*^ and *Osx*^*f/f*^ mice. n=6, data are presented as mean±s.d., unpaired, two-tailed Student’s t test. **f,** 3D reconstruction of L5 vertebrae μCT from 4-week-old *Stat3*^*zic1*^ and *Stat3*^*f/f*^ mice. Scale bar, 100 μm. **g,** Quantification of bone volume/total volume (BV/TV), trabecular number (Tb.N), trabecular thickness (Tb.Th) and trabecular separation (Tb.Sp) of L5 vertebrae in 8-week-old *Stat3*^*zic1*^ and *Stat3*^*f/f*^ mice. n=5 or 7, data are mean±s.d., unpaired, two-tailed Student’s t test. **h,** 3D reconstruction of L5 vertebra (top) and femur (bottom) μCT from 8-week-old *Shn3*^*zic1*^ and *Shn3*^*f/f*^ mice. Scale bar, 100 μm. **i,** Quantification of bone volume/total volume (BV/TV), trabecular number (Tb.N), trabecular thickness (Tb.Th) and trabecular separation (Tb.Sp) of L5 vertebrae (top) and femur (bottom) in 8-week-old *Shn3*^*zic1*^ and *Shn3*^*f/f*^ mice. n=7–8, data are mean±s.d., unpaired, two-tailed Student’s t test. **j,** Immunostaining for PAX1 and ZIC1 in a human endplate specimen showing co-expression of PAX1 and ZIC1. Images are representative of 3 independent experiments. Scale bar, 20 μm. **k,** Flow cytometry analysis of human vSSCs in a human endplate specimen. Plots are representative of 5 independent experiments. **l,** uCT analysis showing 3D reconstruction of human vSSCs and sponge control derived bone organoids 6 weeks after intramuscular transplantation. n=3, scale bar 0.5 mm. **m,** Representative images of Von Kossa staining (left) for mineralized bone and Safranin O staining (right) for cartilage in the bone organoids derived from human vSSCs and sponge control 6 weeks after intramuscular transplantation. n=3. Scale bars, 500 μm. **n,** Flow cytometry analysis of human vSSC-derived cell populations 7 days after intramuscular transplantation. Plots are representative of 2 independent experiments.

**Fig. 5 |:**
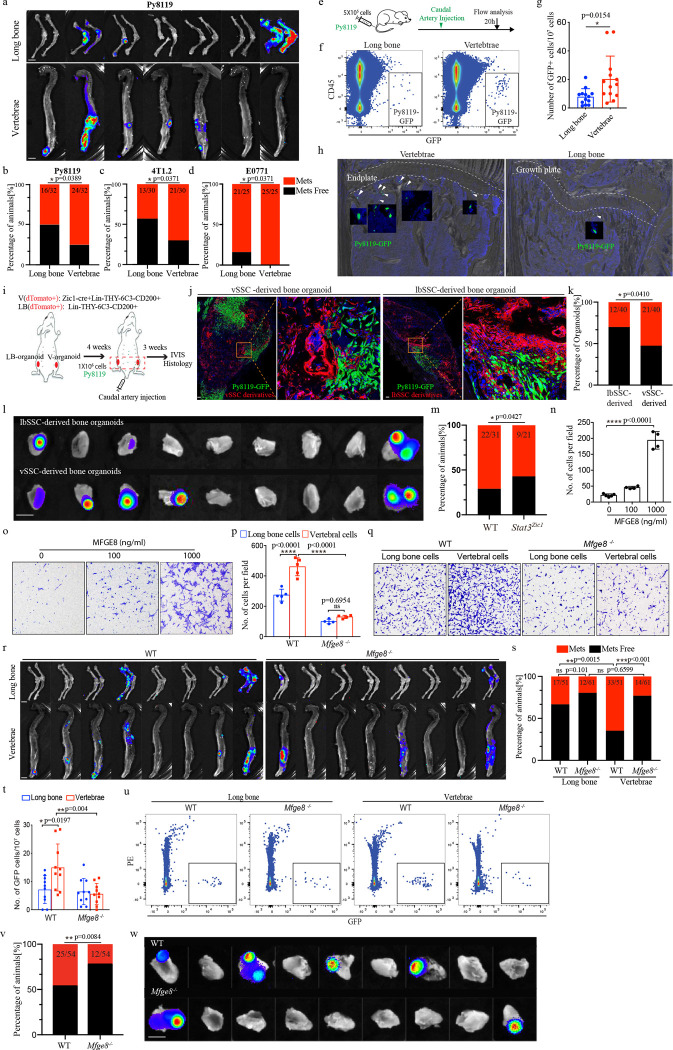
vSSCs drive the preferential metastasis of breast cancer to the vertebrae. **a**, Representative bioluminescence images showing metastasis of PY8119 cells to long bones and vertebrae 4 weeks after injection via the caudal artery. Scale bar, 5 mm. **b-d**, Quantification of metastasis rates to long bones and vertebrae 3–4 weeks after caudal artery injection with PY8119 (**b**, 4 weeks, n=32), 4T1.2 (**c**, 4 weeks, n=30), and E0771 cells (**d**, 3 weeks, n=25). Chi-square test. **e**, Schematic diagram of the early seeding experiment. **f.** Representative flow cytometry plots showing the initially seeding cancer cells in long bones and vertebrae after caudal artery injection. **g,** Quantification of the early seeding cancer cells in vertebrae and long bone 20h after cancer cells injection. n=13, data are mean±s.d., unpaired, two-tailed Student’s t test. **h,** Representative histology images of three independent experiments showing the early seeding cancer cells in vertebrae and long bones. **i,** Schematic diagram of the bone organoid metastasis experiment. **j,** Representative fluorescent histology images showing PY8119 (Green) metastasis to lbSSC-derived and vSSC-derived bone organoids (Red) 3 weeks after cancer cells injection, scale bar 200 μm. **k,** Quantification of the metastasis rate of PY8119 cells to lbSSC-derived and vSSC-derived bone organoids 3 weeks after cancer cells injection. n=40. Chi-square test. **l,** Representative bioluminescence images showing PY8119 metastasis in lbSSC-derived and vSSC-derived bone organoids 3 weeks after cancer cells injection. Scale bar, 5 mm. **m,** Quantification of the metastasis rate of PY8119 cells to WT or *Stat3*^*zic1*^ vertebrae 4 weeks after cancer cells injection. n=31 for WT mice and n=21 for *Stat3*^*zic1*^ mice. Chi-square test. **n&o,** Quantification (**n**) and representative images (**o**) of PY8119 transwell migration in the presence of the indicated concentrations of recombinant MFGE8 in the lower chamber. n=4, data are mean±s.d., one-way ANOVA followed by Tukey’s multiple comparison test. **p&q,** Quantification (**p**) and representative images (**q**) of PY8119 transwell migration in the presence of bone marrow stromal cells in the lower chamber isolated from long bones or vertebrae of 3-week-old WT or *Mfge8*^*−/−*^ mice. n=4 or 5, data are mean±s.d., two-way ANOVA followed by Tukey’s multiple comparison test. **r,** Representative bioluminescence images showing cancer metastasis in long bones and vertebrae of WT and *Mfge8*^*−/−*^ mice 4 weeks after PY8119 cells injection through caudal artery. Scale bar, 5 mm. **s**, Quantification of the metastasis rate to long bones and vertebrae 4 weeks after caudal artery injection with PY8119 cells, n=51 for WT mice and n=61for *Mfge8*^−/−^ mice. Chi-square test. **t,** Quantification of the early seeding of cancer cells in the vertebrae and long bones of WT and *Mfge8*^*−/−*^ mice 20h after cancer cells injection. n=11 for WT and n=10 for *Mfge8*^−/−^ mice, data are mean±s.d. Two-way ANOVA followed by Tukey’s multiple comparison test. **u,** Representative flow cytometry plots showing the early seeding of cancer cells in long bone and vertebrae of WT and *Mfge8*^*−/−*^ mice 20h after cancer cells injection. **v,** Quantification of the metastasis rate of PY8119 cells to WT or *Mfge8*^*−/−*^ vSSC-derived bone organoids 3 weeks after cancer cells injection. n=54. Chi-square test. **w,** Representative bioluminescence images showing metastasis of PY8119 cells to WT or *Mfge8*^*−/−*^ vSSC-derived bone organoids 3 weeks after caudal artery injection. Scale bar, 5 mm.
